# Formulation of Peptide‐Based Nanoparticles Using a Microfluidic Device

**DOI:** 10.1002/psc.70107

**Published:** 2026-06-10

**Authors:** Thania Hammoum, Karidia Konate, Yannick Mousli, Sébastien Deshayes, Eric Vivès, Audrey Nsamela, Prisca Boisguérin

**Affiliations:** ^1^ PhyMedExp, University of Montpellier, INSERM U1046, CNRS UMR9214 Montpellier France; ^2^ Inside Therapeutics Bègles France

**Keywords:** microfluidic, pDNA, peptide‐based nanoparticle, siRNA, WRAP5

## Abstract

Peptide‐based nanoparticles (PBN) have emerged as a promising alternative to lipid nanoparticles (LNP) for nucleic acid delivery and efficient cellular uptake. In this study, we evaluated the formulation of WRAP5 (W‐ and R‐rich amphipathic peptide 5)–based PBN using a microfluidic device and assessed the impact of key process parameters, flow rate ratio (FRR), total flow rate (TFR), and mixing channel design, on nanoparticle characteristics. Across 72 formulations encapsulating small interfering RNA (siRNA) or plasmid DNA (pDNA), dynamic light scattering revealed consistent mean sizes ranging from 50 to 70 nm, with a low polydispersity index (PdI < 0.22), independent of FRR, TFR, or mixer type. Stability studies demonstrated that siRNA‐loaded PBN exhibited moderate size increases during storage at 4°C, whereas pDNA‐loaded PBN remained highly stable for up to 70 days. Biological assays confirmed robust activity: WRAP5:siRNA PBN achieved approximately 50% CDK4 silencing in GIST‐T1 cells, and WRAP5:pDNA PBN mediated efficient mCHERRY expression in HeLa cells, regardless of formulation method or storage duration. These findings highlight the robustness and scalability of WRAP5‐based PBN, contrasting with LNP systems that require stringent control of FRR and TFR, and partially underscore their potential for nucleic acid delivery applications.

## Graphical abstract

A case study using the TAMARA microfluidic device (Inside Therapeutics) was performed for peptide‐based nanoparticles under different operational parameters such as mixing channel geometry, total flow rate, and flow rate ratio. In this context, WRAP5 cell‐penetrating peptide‐based nanoparticles for siRNA or pDNA delivery demonstrated enhanced homogeneity and reproducibility compared to manually performed formulations.

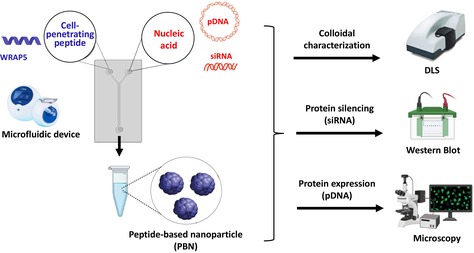

## 1 Introduction

Nonviral nucleic acid delivery systems have become central to the advancement of gene therapy and RNA‐based therapeutics. These systems are designed to protect nucleic acids from degradation and facilitate their transport into target cells, offering safer alternatives to viral vectors [1, 2]. Among the most prominent nonviral carriers are lipid‐based nanoparticles (LNPs), polymeric vectors, peptide‐based nanoparticles (PBN), protein‐based nanoparticles, and inorganic nanoparticles (NP).

In general, NPs are synthetically engineered particles with dimensions around 100 nm, and their physicochemical properties enable them to traverse cellular membranes. Their development has significantly broadened their scope in biomedical applications, including drug and gene delivery, bioimaging, and oncological therapies. NPs are designed to encapsulate therapeutic agents or adsorb them onto their surfaces (e.g., mesoporous silica NPs), contributing to enhanced pharmacokinetics and therapeutic efficacy of the associated drugs.

Especially, LNPs are currently one of the most clinically validated delivery systems, having been successfully used in FDA‐approved therapeutics such as Onpattro (patisiran) [3] and more recently in mRNA vaccines for COVID‐19 [4]. These NPs typically consist of ionizable lipids, helper lipids, cholesterol, and PEG‐lipids. However, LNPs tend to accumulate in the liver, which limits their use for targeting other organs. To address this, researchers are developing LNP composed of biodegradable lipids and surface modifications to redirect those NPs to nonhepatic tissues [5].

In the field of safer delivery systems, PBNs, especially cell‐penetrating peptides (CPPs) [6], offer efficient membrane translocation and minimal immunogenicity [7]. These carriers often rely on electrostatic interactions between cationic amphipathic peptides and negatively charged nucleic acids. Peptides may be used alone or in combination with other compounds to improve stability, targeting, and endosomal escape.

In this context, we have developed a novel class of amphipathic peptides, termed WRAP (W‐ and R‐rich amphipathic peptides, LLRLLRWWWRLLRLL), capable of forming PBN upon incubation with siRNA at a CPP:siRNA molar ratio (MR) of 20 [8]. The initial proof‐of‐concept demonstrated the siRNA transfection efficiency mediated by WRAP peptides in different cancer cell lines, targeting both overexpressed and endogenous proteins. Subsequent evaluations were extended to nine additional cell lines, as well as vascular endothelial cells and human‐induced pluripotent stem cell‐derived cardiomyocytes [9].

The high transfection efficacy observed across diverse cell lines was primarily attributed to the rapid cellular uptake of WRAP‐based PBN (∼15 min), predominantly via direct membrane translocation, with some minor opportunistic use of endocytosis‐dependent mechanisms [10]. This uptake mechanism was elucidated through multiple experimental approaches, including liposome leakage assays to confirm membrane interaction and the use of endocytosis inhibitors and endosomal/lysosomal markers. Notably, fluorescently labeled siRNAs delivered by WRAP‐based PBN accumulated in Dynamin triple‐knockout (KO) cells, which lack functional endocytic pathways, further supporting a nonendocytic mode of entry.

A structure–activity relationship analysis identified WRAP5 as a leading CPP candidate. Comparative studies with lipid‐based transfection reagents, such as Lipofectamine RNAiMAX, revealed that WRAP:siRNA PBN achieve comparable levels of protein silencing without inducing cytotoxicity, as confirmed by clonogenic assays [11]. Finally, in vivo investigations demonstrated effective gene silencing using WRAP:siRNA PBN in two models: luciferase knockdown in a mouse glioblastoma xenograft model [12] and green fluorescent protein (GFP) silencing in transgenic zebrafish expressing GFP in vascular endothelial cells [13].

The development of NPs for nucleic acid delivery (LNP or PBN) relies on a set of critical physicochemical and process parameters that determine their efficacy, stability, and reproducibility. Protocols with standard procedures are needed to enhance homogeneity and reduce batch‐to‐batch variability for clinical applications. Therefore, microfluidic systems allow precise control over parameters such as carrier‐to‐cargo ratio, buffer conditions, and mixing speed, which are critical for achieving optimal NP characteristics and biological activity [14, 15].

Importantly, the physicochemical principles governing NP formation differ substantially between LNP and PBN. Conventional LNP formulation requires an organic solvent to solubilize hydrophobic lipids, followed by a rapid solvent‐exchange process upon mixing with an aqueous phase, which drives lipid self‐assembly into NPs. In contrast, WRAP‐based PBN formation relies exclusively on electrostatic interactions between cationic amphipathic peptides and negatively charged nucleic acids and therefore does not require the presence of an organic phase or solvent exchange. As a result, PBN assembly can be fully achieved through an aqueous‐to‐aqueous formulation process.

Microfluidic devices using mixers such as staggered herringbone mixers and baffle mixers enable rapid and controlled mixing, resulting in NPs with narrow size distributions (80–150 nm depending on their composition) while maintaining a low polydispersity index (PdI < 0.2), a critical parameter for reproducibility and optimal biological performance. For example, the TAMARA microfluidic platform (Inside Therapeutics) was specifically developed for precise organic NP formulation using minimal reagent volumes, typically between 0.2 and 3 mL, making it particularly suited for screening and optimization workflows in research and preclinical development settings [16].

In this study, we assessed the potential benefits of formulating PBN using the TAMARA microfluidic device, as this approach has not, to our best knowledge, been previously reported in the literature. Specifically, WRAP5‐based NPs were formulated with siRNA or pDNA, depending on the type of mixers used (herringbone or baffle), the flow rate ratio (FRR), and the total flow rate (TFR). The activity of the formulated NP was evaluated using cyclin‐dependent kinase 4 (CDK4) silencing (Western blot) and mCHERRY overexpression (microscopy).

## 2 Results and Discussion

### 2.1 Formulation of the WRAP Nanoparticles Using the TAMARA Microfluidic Device

**FIGURE 1:**
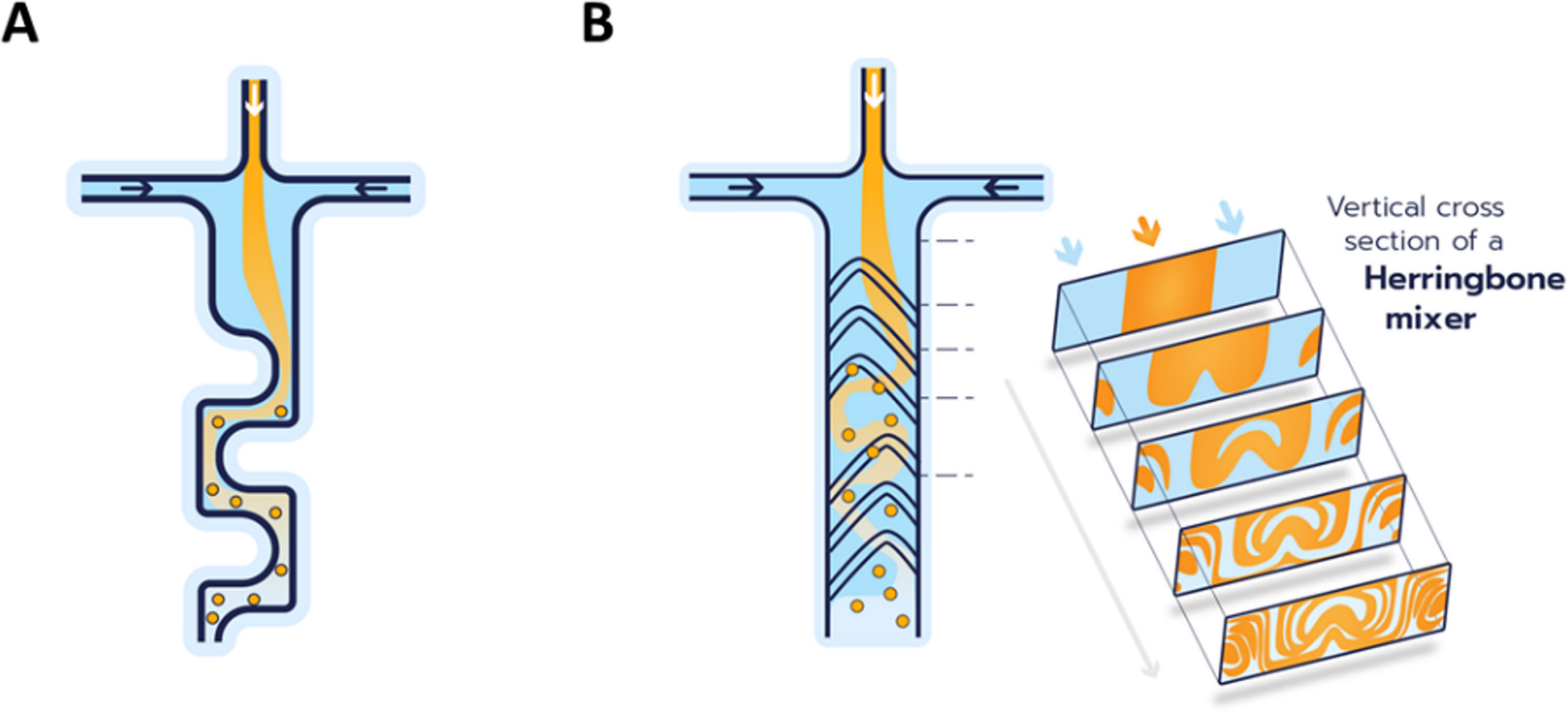
Schematic illustration of the two mixing channels used in this study. (A) The baffle mixer enables accelerated mixing through the generation of vortices. This mechanism facilitates the formation of smaller nanoparticles while supporting higher total flow rates compared to herringbone‐based mixing strategies. (B) The herringbone mixer employs a distinctive mixing mechanism that creates microvortices within a rigid microchannel, into which two reagent streams are introduced. These vortices cause the liquid phases to repeatedly fold over one another, significantly enhancing the interfacial surface area and thereby promoting efficient mixing between the two phases. These schematics are provided for illustrative purposes only and do not represent a scale‐accurate model of the microfluidic device.

The TAMARA microfluidic device (Inside Therapeutics) features two distinct mixing channels integrated on a reusable chip: a herringbone mixer and a baffle mixer arranged in a head‐to‐tail configuration (Figure 1). The herringbone mixer relies on chevron‐shaped grooves that induce chaotic advection by repeatedly stretching and folding fluid streams, thereby enabling efficient mixing under laminar flow conditions. This design makes it particularly suitable for small‐volume formulations and has led to its widespread use in LNP production due to its robustness and reproducibility. In contrast, the baffle mixer employs internal obstructions to redirect fluid flow and generate vortical structures; this configuration performs optimally at elevated flow rates but exhibits reduced mixing uniformity under low Reynolds number conditions [17].

In typical LNP microfluidic formulations, efficient hydrodynamic focusing and rapid mixing are achieved at Reynolds numbers (Re) in the range of approximately 1–100, depending on device geometry and channel design, where chaotic advection or Dean vortices promote efficient solvent exchange and NP self‐assembly [18, 19]. In the present study, because both input phases consist exclusively of an aqueous solution (5% glucose), the estimated Reynolds numbers fall within a lower regime (Re ≈0.1–5) across the tested TFR range [20, 21]. Although this regime lies below that commonly employed for LNP formulation, it remains compatible with effective mixing in microchannels specifically designed for laminar flow. Consequently, at low Reynolds numbers, mixing is mainly ensured by the herringbone channel, whereas the contribution of the baffle mixer becomes more prominent at higher TFRs, where secondary flows (e.g., Dean vortices), arising from channel obstructions, enhance mixing efficiency.

Two other parameters deserve consideration in addition to the mixing channels. The first one is the flow rate ratio (FRR), defined as the ratio between the flow rates of two (or more) fluids entering a microfluidic device, typically through separate channels. Specifically, FRR refers to the proportion between the organic phase (typically containing the polymer or lipid for NP formation) and the aqueous phase (usually carrying the cargo). The FRR governs the spatial arrangement of fluids, interface dynamics, and the efficiency of particle formation. The optimization of the flow rate ratio has been demonstrated to enhance mixing by increasing interfacial area and promoting chaotic advection or vortex formation. The second parameter in microfluidic NP synthesis is the TFR. The TFR governs the overall speed at which fluids, typically an organic phase containing dissolved precursors and an aqueous phase, are introduced and mixed within the microchannel. Its function is intimately associated with the processes of mixing dynamics, NP nucleation, growth, and, in the final analysis, the physicochemical properties of the NPs that are produced [22]. In general, small‐sized LNP can be obtained at low aqueous to organic FRR and at high TFR [18, 23].

In this study, we evaluate the impact of the two mixing channel designs (baffle and herringbone), three FRRs (1:1, 3:1, and 5:1), and three different TFRs (1.5, 5, and 8 mL/min) on WRAP‐based PBN formulations. Unlike conventional organic polymer or lipid formulations, all experiments employed the same aqueous phase (5% glucose) in both channels: (1) for the WRAP5 solution and (2) for the siRNA or pDNA cargo to be encapsulated. Each condition was tested in duplicate, resulting in a total of 72 formulations. Mean sizes (d.nm) and polydispersity index (PdI) were determined using dynamic light scattering (DLS) for all of them. Representative data are summarized in Tables 1 and 2 for an FRR of 1:1 and the three analyzed TFRs. All other conditions (FRR of 3:1 and 5:1) are given in the Supplementary Information (Tables S1 and S2).

**TABLE 1:** Characterization of the WRAP5:siRNA PBN formulated at an FRR of 1:1 by DLS.

Time	Condition	TFR (mL/min)	Mean size (d.nm)	PdI
d0	W5:siRNA (B)	1.5	54.2 ± 1.3	0.204 ± 0.006
		5	57.2 ± 1.9	0.185 ± 0.017
		8	57.5 ± 2.8	0.202 ± 0.016
	W5:siRNA (H)	1.5	64.0 ± 5.2	0.204 ± 0.043
		5	61.7 ± 1.2	0.175 ± 0.007
		8	67.4 ± 2.3	0.221 ± 0.023
	Upscale‐W5:siRNA (B)	5	63.4 ± 0.3	0.237 ± 0.012
	Upscale‐W5:siRNA (H)	5	99.9 ± 0.7	0.171 ± 0.004
	Upscale‐W5:siRNA (M)	—	77.5 ± 2.3	0.341 ± 0.018
d7	W5:siRNA (B)	1.5	96.4 ± 10.5	0.228 ± 0.012
		5	94.2 ± 4.7	0.214 ± 0.015
		8	94.2 ± 3.2	0.217 ± 0.008
	W5:siRNA (H)	1.5	100.4 ± 3.5	0.211 ± 0.013
		5	117.9 ± 3.9	0.239 ± 0.012
		8	113.0 ± 8.4	0.244 ± 0.002
	Upscale‐W5:siRNA (B)	5	97.9 ± 0.8	0.183 ± 0.008
	Upscale‐W5:siRNA (H)	5	88.9 ± 2.9	0.220 ± 0.010
	Upscale‐W5:siRNA (M)	—	80.2 ± 2.1	0.231 ± 0.016

*Note:* All WRAP5:siRNA complexes were formed at MR = 20 using a siRNA concentration of 500 nM. All formulations were performed in a filtered aqueous solution of 5% glucose. DLS measurements were performed on the same day of formulation (d0) and at 7 days postformulation (d7). All formulations were performed at a FRR of 1:1 (final volume of 0.5 mL). *n* = 2 independent formulations (three measures per run). Upscale conditions correspond to formulation performed at a higher volume (1.5 mL).

Abbreviations: B, baffle; H, herringbone; M, manual; PdI, polydispersity index.

**TABLE 2:** Characterization of the WRAP5:pDNA PBN formulated at an FRR of 1:1 by DLS.

Time	Condition	TFR (mL/min)	Mean size (d.nm)	PdI
d0	W5:pDNA (B)	1.5	52.5 ± 0.5	0.156 ± 0.011
		5	50.2 ± 0.9	0.175 ± 0.019
		8	49.3 ± 2.5	0.191 ± 0.034
	W5:pDNA (H)	1.5	47.9 ± 1.4	0.184 ± 0.027
		5	56.6 ± 1.6	0.180 ± 0.011
		8	53.8 ± 2.1	0.183 ± 0.019
	Upscale‐W5:pDNA (B)	1.5	48.7 ± 1.3	0.196 ± 0.017
	Upscale‐W5:pDNA (H)	1.5	49.9 ± 0.6	0.150 ± 0.002
	Upscale‐W5:pDNA (M)	—	56.1 ± 1.9	0.219 ± 0.026
d7	W5:pDNA (B)	1.5	51.8 ± 2.6	0.156 ± 0.016
		5	51.3 ± 0.7	0.173 ± 0.013
		8	50.4 ± 2.2	0.148 ± 0.006
	W5:pDNA (H)	1.5	54.9 ± 0.4	0.150 ± 0.008
		5	57.3 ± 1.0	0.160 ± 0.009
		8	55.8 ± 2.8	0.175 ± 0.028
	Upscale‐W5:pDNA (B)	1.5	47.0 ± 1.3	0.133 ± 0.005
	Upscale‐W5:pDNA (H)	1.5	52.6 ± 3.1	0.161 ± 0.036
	Upscale‐W5:pDNA (M)	—	59.1 ± 1.0	0.216 ± 0.024

*Note:* All WRAP5:pDNA complexes were formulated at CR = 3 using a pDNA concentration of 5.2 nM. All formulations were performed in a filtered aqueous solution of 5% glucose. DLS measurements were performed on the same day of formulation (d0) and at 7 days postformulation (d7). All formulations were performed at a FRR of 1:1 (final volume of 0.5 mL). *n* = 2 independent formulations (three measures per run). Upscale conditions correspond to formulation performed at a higher volume (1.5 mL).

Abbreviations: B, baffle; H, herringbone; M, manual; PdI, polydispersity index.

At a constant molar ratio (MR) of 20 for the WRAP5:siRNA, we observed the formation of PBN with mean sizes between 55 and 67 nm with PdI values between 0.18 and 0.22 when measured the same day of the formulation (d0). In detail, we could not observe any influence of the mixing channel used, of the different mixing parameters (FRRs and TFRs) on PBN sizes and homogeneity (Tables 1 and S1).

These results are in accordance with previous findings showing by transmission electron microscopy (TEM) that WRAP‐based siRNA PBNs form a heterogeneous NP population composed of elongated “beads‐on‐a‐necklace” assemblies (~100 nm) coexisting with smaller discrete particles (~20–30 nm). The presence of such mixed‐size populations was further supported by DLS measurements, which revealed only minor differences between intensity‐ and number‐weighted size distributions (data not shown). Finally, considering the experimental differences between DLS measurements on solvated samples and TEM imaging of dried samples, the size distributions obtained by both techniques are in good agreement for the two WRAP NP formulations.

The WRAP5:pDNA PBN condition at a constant charge ratio (CR) of three revealed mean sizes between 48 and 57 nm with PdI values between 0.16 and 0.19 when measured the same day as the formulation (d0) (Table 2). As shown above for the WRAP5:siRNA, we could not observe any influence of the mixing channel used, nor of the different mixing parameters (FRRs and TFRs), on PBN sizes and homogeneity (Tables 2 and S2). However, these results highlight that PBN encapsulating pDNA were generally smaller (52 ± 4 nm) than those loaded with siRNA (106 ± 12 nm) (Tables 1 and 2).

Based on the fact that there was no effect of the FRR and TFR on PBN formulation, we selected the FRR parameters of 1:1 for the upscaled batch formulations, as this condition reflects the previously used manual formulation [8]. Furthermore, we have used a TFR of 5 for the WRAP5:siRNA and a TFR = 1.5 for the WRAP5:pDNA PBN, respectively. These upscaled batches were compared to a manually formulated one. DLS measurements revealed no important differences in the previously measured low volume (0.5 mL) compared to the upscaled (1.5 mL) or manual formulations (1.5 mL) (Tables 1 and 2), showing the formulation volume does not impact the NP colloidal features.

### 2.2 Evolution of WRAP5 Nanoparticles Formulated With a Microfluidic Device Over Time

A pivotal aspect in the development of NP pertains to their temperature‐dependent stability. For instance, the stability of mRNA lipid NP at cool or ambient temperatures is a concern for mRNA vaccines [24]. Therefore, we evaluated the storage of WRAP‐based PBN under mild conditions. In detail, the upscaled formulations were stored at 4°C, and at designated time points, a portion of the solution was extracted for DLS analysis.

**FIGURE 2:**
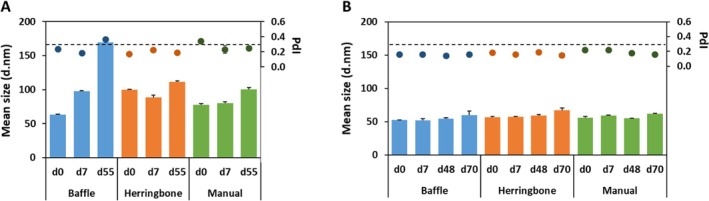
Evaluation of the PBN stability upon storage at 4°C measured by DLS. (A) WRAP5:siRNA complexes were formulated at MR = 20 with a siRNA concentration of 500 nM using TAMARA's mixing devices (baffle or herringbone, FRR 1:1 and TFR 5) or using a manual formulation. DLS measurements were performed on the same day of formulation (d0), at 7 days (d7) or 55 days (d55) postformulation. (B) WRAP5:pDNA complexes were formulated at CR = 3 with a pDNA concentration of 5.2 nM using TAMARA's mixing devices (baffle or herringbone, FRR 1:1 and TFR 1.5) or using manual formulation. DLS measurements were performed on the same day of formulation (d0), at 7 days (d7), at 48 days (d48), or 70 days (d70) postformulation. For all formulations, the mean size and the PdI values are provided.

First, we analyzed the effect of WRAP5:siRNA PBN stored at 4°C during 7 days (d7) as previously published [8]. We observed by DLS measurements that the mean sizes of the different PBN increased slightly, from ~60 to ~100 nm, with PdI values still below 0.25 (Table 1). If the “upscaled” PBNs were stored for a longer time (up to 55 days postformulation), the observed mean size stayed stable at a value around 100 nm (Figure 2A and Table S3). Only the formulation performed using the baffle mixer exhibited reduced stability in comparison to those executed with the herringbone or manual methods. This observation was substantiated by the augmented mean size (> 150 nm).

Compared to the slightly increased size of the WRAP5:siRNA PBN after a storage period of 7 days at 4°C, the mean sizes and PdI values of WRAP5:pDNA PBN did not change (51 nm–57 nm/0.150–0.180, respectively).

Moreover, for the WRAP5:pDNA “upscaled” formulations stored at 4°C for several days (d7), we could observe an impressive stability of the PBN (Tables 2 and S4). No significant changes in their mean sizes (50 nm–70 nm) and in their PdI values (> 0.3) were observed when measured by DLS at 7, 48, and 70 days postformulation (Figure 2B). Furthermore, no significant differences were observed between the formulation conditions (baffle vs. herringbone vs. manual).

### 2.3 Transfection Efficiency of WRAP5:siRNA Nanoparticles Formulated With a Microfluidic Device

**FIGURE 3:**
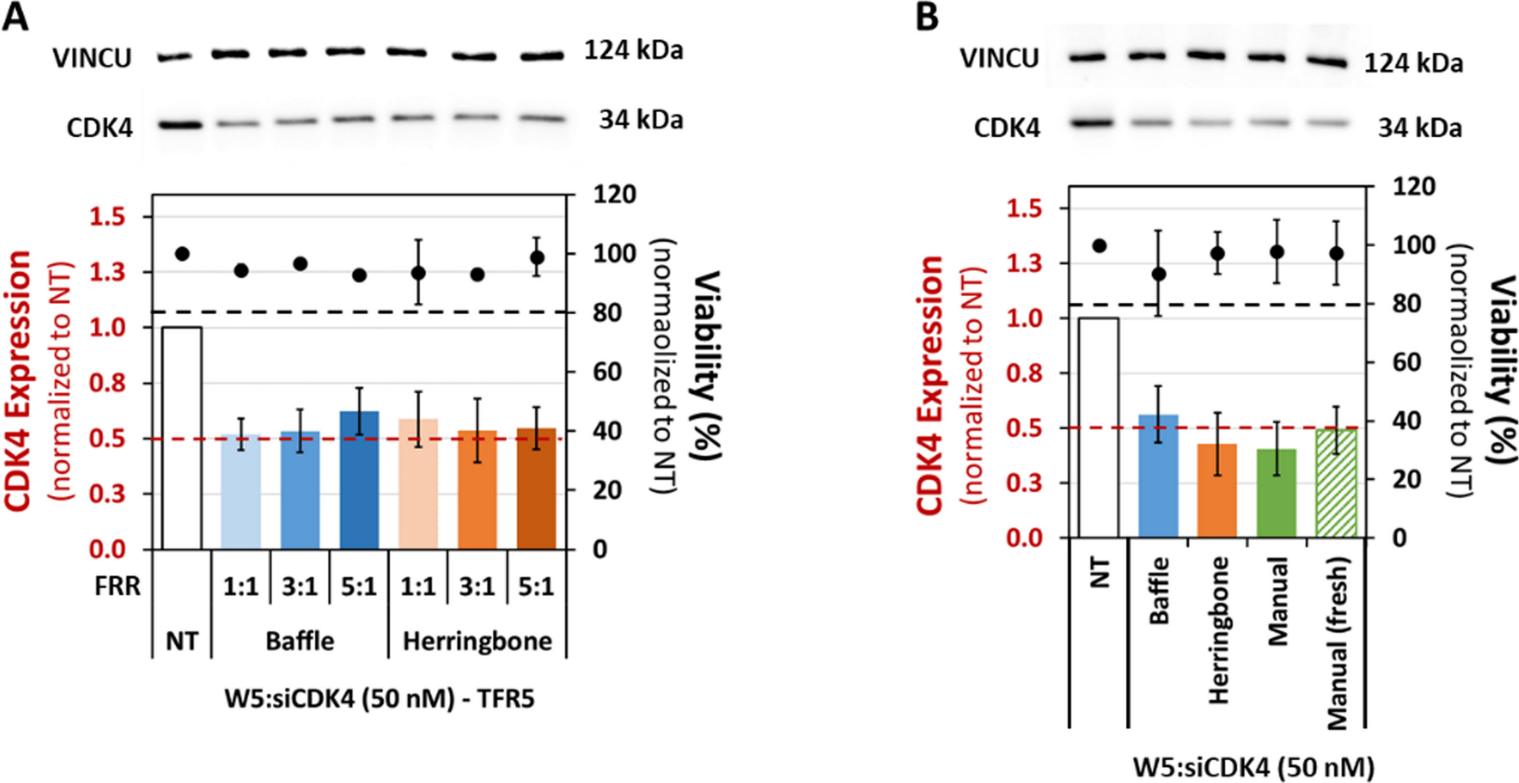
Evaluation of CDK4 silencing using WRAP5:siRNA nanoparticles. (A) WRAP5 PBN encapsulating an siRNA targeting the CDK4 protein was formulated at a constant TFR of 5 and different FRR, as indicated, using the baffle or herringbone mixing devices. These PBN induced 50% silencing of the CDK4 protein at a final siRNA concentration of 50 nM, as revealed by Western blot after a 24 h incubation. (B) Upscale batches of WRAP5:siCDK4 PBN formulated using the baffle or herringbone mixing devices (TFR of 5 and FRR of 1:1) or prepared manually were stored for 55 days at 4°C. These formulations, as well as a freshly prepared one, were used to transfect GIST‐T1 cells. Western blot after a 24 h incubation revealed that these PBN induced 50% silencing of the CDK4 protein at a final siRNA concentration of 50 nM.

For a biological readout, we have encapsulated a siRNA targeting cyclin‐dependent kinase 4 (CDK4) that was previously used in human glioblastoma (U87) and gastrointestinal mesenchymal tumor (GIST‐T1) cell lines [8, 25]. The PBN, which was generated using the baffle or herringbone mixers at a constant TFR of 5, and three different FRRs (1:1, 3:1, and 5:1) were used to compare CDK4 silencing in GIST‐T1 cells (1.5 h PBN incubation in serum‐free medium followed by 24 h incubation in serum‐containing medium). In detail, formulations stored at 4°C during four different time points (d14, d21, d29, or d55) were used to evaluate the effect of the storage condition on CDK4 silencing by Western blot. As the quantification of the Western blots revealed the same CDK4 knockdown for all storage periods, we pooled the results into one graphical representation (Figure 3A). At a final siCDK4 concentration of 50 nM, we observed a 50% decrease in CDK4 expression (red dotted line) compared to the nontreated cells (NT) for the six WRAP5:siRNA formulations. Because the different FRRs used did not impact the PBN sizes (see Tables 1 and S1), we did not observe differences in the silencing properties of these formulations. The level of CDK4 silencing was the same as that previously reported [25].

Afterward, we evaluated the three “upscaled” formulations generated using the baffle or herringbone mixing devices or a manual procedure, which were stored at 4°C for 55 days. First, we confirmed that the mean sizes and PdI values were similar to those observed at 7 days postformulation by DLS measurements (Table S5). Then, GIST‐T1 cells were transfected using the same incubation conditions as described above, along with a freshly manual formulation. Subsequent Western blot analysis revealed a significant reduction in CDK4 expression in comparison to the nontreated cells for all WRAP5:siRNA formulations at a final siRNA concentration of 50 nM (Figure 3B). Here, again, CDK4 silencing of around 50% (red dotted line) was comparable to that observed previously [8, 25].

More importantly, for all conditions, cell viability was not impacted and was similar to the nontreated cells (100% ± 20%, black dotted line in Figure 3). However, for the upscaled WRAP5:siRNA formulation performed with the baffle mixer (Figure 3B), a modest correlation was noticed between a less significant CDK4 knockdown and a slight impact on cell viability, which could be associated with the larger size (~170 nm) of the used PBN (Figure 1A and Tables 1 and S1).

### 2.4 Transfection Efficiency of WRAP5:pDNA Nanoparticles Formulated With a Microfluidic Device

Regarding the biological activity of the WRAP5:pDNA, we used the mCHERRY plasmid to quantify the transfection performance of the PBN formulated with the different parameters—baffle, herringbone, or manual (see Table S6 for DLS measurements). The transfection using the “upscaled” batches of WRAP5:pDNA‐mCHERRY stored for 48 days at 4°C was performed in HeLa cells using a final pDNA quantity of 1 μg (1.5 h PBN incubation in serum‐free medium followed by 24 h incubation in serum‐containing medium). After the transfection, HeLa cells were fixed with a 2% paraformaldehyde solution and mounted on glass slides.

**FIGURE 4:**
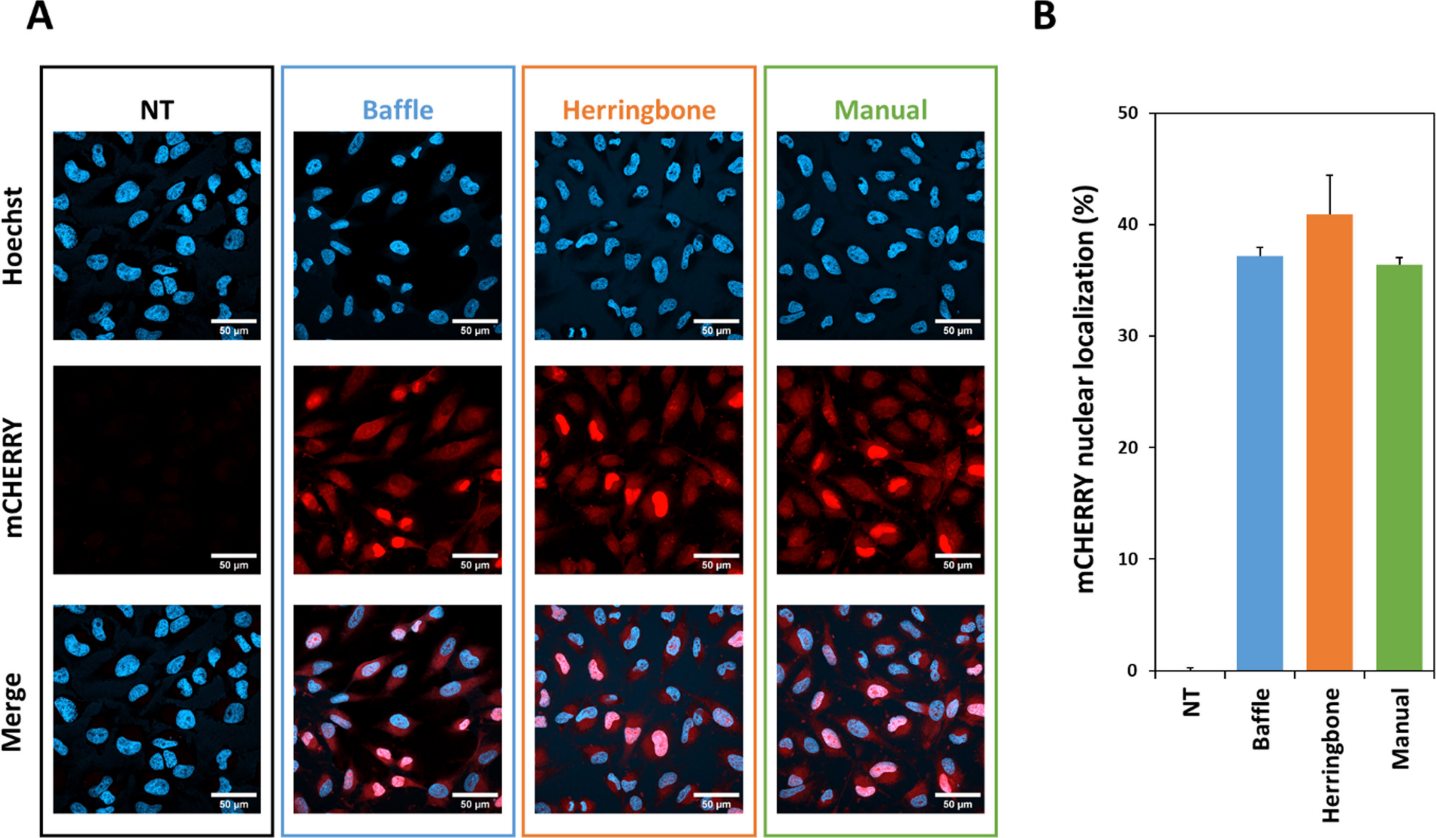
Evaluation of mCHERRY expression using WRAP5:pDNA nanoparticles. (A) Representative images show the individual (blue = Hoechst dye and red = mCHERRY) as well as the merged channels of HeLa cells incubated with WRAP5:pDNA‐mCHERRY PBN, formulated with the baffle or the herringbone devices, compared with a manually formulated batch or nontreated cells (NT). White bar = 50 μm. (B) Graphical representation of the mCHERRY nuclear expression. For each condition, the nuclear red fluorescence (mCHERRY) and the nuclear blue fluorescence (Hoechst) were quantified using ImageJ. To calculate the percentage of nuclear‐positive HeLa cells, the red fluorescence values were normalized to the corresponding nuclei labeled with the Hoechst dye. Formulation conditions: Upscale batches of WRAP5 PBN encapsulating a plasmid to express mCHERRY were formulated using the baffle or herringbone mixing devices (TFR of 1.5 and FRR of 1:1) or prepared manually and were stored for 48 days at 4°C. These formulations were applied to HeLa cells. After 24 h of incubation, cells were fixed and mounted on glass slides.

To quantify the mCHERRY transfection efficacy, more than four arbitrarily selected regions of each condition were imaged using a confocal fluorescence microscope (Figure 4A). As the plasmid encodes an mCHERRY protein containing a nuclear localization sequence (NLS), the transfection efficiency could be easily quantified using ImageJ and normalized through the overall nuclear Hoechst (blue) signal (Figure 4B). These quantifications revealed that more than 35% of all HeLa cells have a nuclear mCHERRY expression level independent of the formulation conditions (baffle, herringbone, or manual).

However, we could clearly see a red fluorescent signal in the cytoplasm. As the cells were fixed at a distinct time point (24 h after transfection), the images reflect not only the mCHERRY proteins located in the nucleus due to their NLS sequence but also those that are currently being translated in the cytoplasm. For this reason, we believe that the transfection efficiency of WRAP5:pDNA‐mCHERRY has been underestimated and that all HeLa cells may have been successfully transfected.

## 3 Conclusion

For the first time, we report the formulation of PBNs using a microfluidic device operating under an aqueous‐to‐aqueous mixing regime, whereas microfluidic technologies are conventionally applied to lipid nanoparticle (LNP) production through aqueous‐to‐organic solvent exchange.

The vast majority of microfluidic formulations involving peptides concern peptide‐decorated or peptide‐loaded NP, typically polymeric or lipid‐based systems relying on an aqueous‐to‐organic solvent exchange mechanism [26, 27, 28]. In contrast, examples of peptide‐only nanocarriers produced by microfluidic approaches remain scarce. Notably, Ni et al. [29] described the fabrication of ultrashort peptide NP encapsulating curcumin using a custom microfluidic device, resulting in particles smaller than 100 nm with low polydispersity (PdI < 0.2). Li et al. [30] reported that insect cuticle–derived peptides spontaneously form hollow nanocapsules via a single‐step solvent‐exchange process, where a concentration gradient created by mixing water and acetone drives peptide localization and self‐assembly. However, similarly to LNP formulation, this strategy relied on an aqueous‐to‐organic solvent exchange process.

In this study, WRAP5‐based PBNs were formulated using the TAMARA microfluidic device for siRNA and pDNA delivery. Comprehensive biophysical and biological characterization demonstrated that all tested formulations consistently exhibited stable physicochemical properties. Neither the type of mixing channel (baffle versus herringbone) nor variations in flow rate ratio (FRR) and TFR had a significant impact on particle size or NP homogeneity. Both siRNA‐ and pDNA‐loaded PBNs maintained colloidal stability during storage at 4°C, with siRNA complexes showing a moderate size increase over time, whereas pDNA complexes remained highly stable. Biological assays confirmed robust transfection efficiency: WRAP5:siRNA PBN achieved ~50% CDK4 silencing in GIST‐T1 cells, and WRAP5:pDNA PBN mediated efficient mCHERRY expression in HeLa cells, independent of formulation method or storage duration. These findings demonstrate that the TAMARA microfluidic device enables reproducible, scalable PBN formulation without compromising stability or biological activity.

Microfluidic devices are generally regarded as highly sensitive processes mainly used for the formulation of LNP. In contrast to PBN, previous studies have shown that increasing the FRR, typically by raising the aqueous‐to‐organic phase ratio, accelerates solvent exchange, leading to smaller and more homogeneous LNP [19]. Similarly, higher TFR values enhance mixing intensity and reduce particle size variability, although excessively high flow rates may compromise encapsulation efficiency and stability [31]. These dependencies necessitate precise control of FRR and TFR for reproducible LNP formulation, especially in clinical applications. A similar set of results concerning the impact of FRR and TFR on NP formulation has been observed for polymeric NPs based on poly (lactic‐co‐glycolic acid) (PLGA) formulated with the TAMARA microfluidic device [32].

The TAMARA microfluidic device enables the reproduction of manually prepared formulations while providing precise control over formulation parameters, without compromising NP stability or biological activity [26, 27, 28]. The results of this study demonstrate that WRAP5‐based NPs are largely insensitive to variations in flow rate ratio (FRR) and TFR, as evidenced by the consistent particle sizes obtained across all tested conditions.

When compared with previously reported manually formulated WRAP5‐based PBN loaded with siRNA (Konate et al. [8]: 80.0 ± 4.9, PdI = 0.29 ± 0.05/Konate et al. [11]: 81 ± 29 nm, PdI = 0.28 ± 0.02/Konate et al. [25]: 98 ± 15 nm, PdI = 0.24 ± 0.05) or pDNA (Faria et al. [33]: 272 ± 10.7 nm, PdI = 0.24 ± 0.02), we observed only slight differences in size and polydispersity, accompanied by a generally lower batch‐to‐batch variability. It is important to note that the manual formulation of pDNA‐loaded PBNs described by Faria et al. [33] relied on a markedly different protocol, involving the addition of a peptide solution to plasmid DNA followed by vortexing, which may contribute to the observed discrepancies.

However, we observed a more pronounced variability among manually formulated batches depending on the experimenter's level of experience. In this regard, Table 3 summarizes unpublished DLS data obtained from WRAP5:siRNA formulations prepared by three different experimenters depending on their expertise (beginner vs. expert), highlighting the impact of operator‐dependent factors on batch‐to‐batch reproducibility.

**TABLE 3:** Examples of WRAP5:siRNA formulations depending on the experimenters and their level of expertise.

Experimenter	Expertise	Mean size (d.nm)	PdI	*n*
1	Beginner	183.7 ± 82.9	0.348 ± 0.094	3
	Expert	78.0 ± 13.9	0.232 ± 0.026	4
2	Beginner	151.2 ± 69.2	0.300 ± 0.045	4
	Expert	82.4 ± 9.9	0.251 ± 0.013	4
3	Beginner	140.7 ± 135.5	0.388 ± 0.084	3
	Expert	80.1 ± 8.6	0.257 ± 0.033	6

In this context, the use of a microfluidic platform such as TAMARA offers a clear advantage by significantly improving reproducibility in PBN production and minimizing variability arising from external factors such as operator handling, material differences, or procedural inconsistencies, limitations that are inherently associated with manual formulation approaches. Overall, the robustness of this microfluidic system simplifies scale‐up and reduces the need for stringent parameter optimization, thereby providing a substantial advantage over conventional LNP‐based formulation strategies for nucleic acid delivery.

## Funding

This work was supported by the Agence Nationale de la Recherche (21‐CE18‐0022‐01), La Ligue nationale contre le cancer, INSERM, CNRS, and the University of Montpellier.

## Conflicts of Interest

The authors declare no conflicts of interest.

## Supporting information


**Table S1:** Characterization of the WRAP5:siRNA PBNs at d0 by DLS.
**Table S2:** Characterization of the WRAP5:pDNA PBNs at d0 by DLS.
**Table S3:** Characterization of the WRAP5:siRNA PBNs at d7 by DLS.
**Table S4:** Characterization of the WRAP5:pDNA PBNs at d7 by DLS.
**Table S5:** Characterization of the WRAP5:siRNA “upscale” PBNs at d55 by DLS.
**Table S6:** Characterization of the WRAP5:pDNA PBNs at d48 and d70 by DLS.

## Data Availability

The data that support the findings of this study are available from the corresponding author upon reasonable request.
