# Supplementary material for: Formulation of Peptide‐Based Nanoparticles Using a Microfluidic Device
Source: J Pept Sci. 2026 Jun 10;32(7):e70107. doi: 10.1002/psc.70107 (PMC13250954; doi:10.1002/psc.70107)
Supplement: Supplementary file 1 — Table S1: Characterization of the WRAP5:siRNA PBNs at d0 by DLS. Table S2: Characterization of the WRAP5:pDNA PBNs at d0 by DLS. Table S3: Characterization of the WRAP5:siRNA PBNs at d7 by DLS. Table S4: Characterization of the WRAP5:pDNA PBNs at d7 by DLS. Table S5: Characterization of the WRAP5:siRNA “upscale” PBNs at d55 by DLS. Table S6: Characterization of the WRAP5:pDNA PBNs at d48 and d70 by DLS. [file PSC-32-e70107-s001.docx]

**MATERIALS AND METHODS:**

**Materials**

WRAP5 peptide (LLRLLRWWWRLLRLL) was synthesized at the SynBio3 platform (IBMM Montpellier), and the crude product was purified in-house following a qualitative analysis by HPLC/MS (∼95% purity). WRAP5 stock solutions were prepared at 500 µM and stored at 4 °C.

The CDK4 siRNA sequence (5′-CAG-AUC-UCG-GUG-AAC-GAU-GdTdT-3′) was purchased from Eurogentec. The siRNA stock solution (100 µM) was prepared in RNase-free water, aliquoted, and stored at -20°C.

The pmCherry-C1 mCherry-NLS (mCHERRY pDNA) was a gift from Dyche Mullins (Addgene plasmid #58476).^1^ The mCHERRY pDNA stock solution (2 µg/µL) was stored at -20°C.

**WRAP5-based PBN formulation**

Peptide-based nanoparticles (PBN) were formulated in a filtered aqueous solution of 5 % (w/v) glucose (Sigma‑Aldrich) by mixing WRAP5 peptide and nucleic acid (siRNA or pDNA) solutions using the TAMARA® microfluidic formulation device (Inside Therapeutics). Mixing was performed with reusable COC (Topas) microfluidic chips integrating two distinct mixer geometries, a herringbone mixer and a baffle mixer, arranged in a head‑to‑tail configuration.

All WRAP5 peptide solutions, as well as siRNA and pDNA solutions, were prepared in the same filtered 5 % glucose aqueous solution. This formulation medium was selected to ensure physiological osmolarity while maintaining optimal solubility of both peptides and nucleic acids, as described previously for the CADY:siRNA PBN.^2^ In addition, glucose‑based solutions are fully compatible with downstream biological applications, including in vitro transfection assays and in vivo administration.

Because glucose moderately increases the viscosity of aqueous solutions, the physicochemical parameters specific to a 5 % glucose solution were explicitly implemented in the TAMARA device settings. At 20 °C, this solution exhibits a dynamic viscosity of approximately 1,200 µPa·s, with a temperature sensitivity of ~30 µPa·s per °C. The solvent molar volume was set to 18.5 cm³·mol⁻¹, and all formulations were performed under a constant input pressure of 7 bar. To avoid viscosity‑related variability, all experiments were conducted at a constant and controlled temperature.

Formulations were prepared at final volumes of 500 µL or 1,500 µL (upscaling) by systematically varying the total flow rate (TFR) and flow rate ratio (FRR) using both mixer geometries (baffle and herringbone) at room temperature.

***WRAP5:siRNA formulations:*** For all conditions, a constant final molar ratio (MR) of WRAP5:siRNA = 20:1 was maintained. Dedicated stock solutions were prepared for each FRR condition:

- FRR 1:1: 1.0 µM siRNA / 20 µM peptide
- FRR 3:1: 0.7 µM siRNA / 40 µM peptide
- FRR 5:1: 0.6 µM siRNA / 60 µM peptide

Comparative manual formulations were prepared by volume‑to‑volume mixing (FRR 1:1), while maintaining the same WRAP5:siRNA molar ratio (20:1).

***WRAP5:pDNA***: For all conditions, a constant final charge ratio of WRAP5:pDNA = 3:1 was maintained. To this end, dedicated stock solutions were prepared for each FRR condition: FRR 1:1 (0.01 µM pDNA and 60 µM peptide), FRR 3:1 (0.02 µM pDNA and 40 µM peptide), and FRR 5:1 (0.03 µM pDNA and 36 µM peptide). Comparative formulations made by a volume:volume (FRR1:1) hand mixing were also formulated respecting the charge ratio (WRAP5:spDNA) = 3:1.

***WRAP5:pDNA formulations***: For all conditions, a constant final charge ratio (CR) of WRAP5:pDNA = 3:1 was maintained. Dedicated stock solutions were prepared for each FRR condition:

- FRR 1:1: 0.01 µM pDNA / 60 µM peptide
- FRR 3:1: 0.02 µM pDNA / 40 µM peptide
- FRR 5:1: 0.03 µM pDNA / 36 µM peptide

Manual formulations were prepared at FRR 1:1 by volume mixing while preserving the same WRAP5:pDNA charge ratio (3:1).

For all microfluidic runs, nucleic acid solutions (siRNA or pDNA) were loaded into the “aqueous” manifold and peptide solutions into the “organic solvent” manifold, although both streams consisted exclusively of an aqueous glucose solution.

Between formulations, microfluidic chips were thoroughly cleaned using the TAMARA® integrated cleaning procedure to ensure consistent performance and reproducibility across runs.

**Dynamic light scattering (DLS)**

The mean particle size (Z‑average diameter) and polydispersity index (PdI) of WRAP5-based nanoparticles were determined by dynamic light scattering using a Zetasizer Nano ZS (Malvern Instruments) equipped with a 532 nm laser. Measurements were performed at a backscattering angle of 173° and at 25°C after an equilibration period of 2-5 min. All measurements were performed in triplicate, with three runs recorded for each sample.

The Z‑average diameter corresponds to the intensity‑weighted mean hydrodynamic size, while the PdI provides an estimate of the particle size distribution homogeneity. A perfectly monodisperse sample would exhibit a single defined size with a PdI value close to zero. For PBN, a mean diameter in the range of 50–100 nm combined with a PdI below 0.3 is generally considered indicative of a homogeneous nanoparticle population.

For the time-dependent stability acquisition, samples were stored at 4°C in the DLS cuvette.

**Cell culture conditions.**

The GIST‑T1 cell line, harboring a primary KIT exon 11 Δ560–578 in‑frame deletion, was obtained from Cosmo Bio.^3,4^ The HeLa cell line was purchased from ATCC.

Both cell lines were cultured in Dulbecco’s Modified Eagle’s Medium (DMEM with 10% fetal bovine serum (FBS, Sigma-Aldrich) and 1% penicillin-streptomycin (P/S) (= complete medium). Cells were passaged (Sigma-Aldrich) using Gibco™ 0.05% Trypsin‑EDTA (Thermo Fisher Scientifics) with phenol red and maintained in a humidified incubator at 37 °C with 5% CO₂. All cell cultures were routinely tested every month and confirmed to be mycoplasma‑free using the MycoAlert™-PLUS Kit (Lonza).

**Western blotting.**

For Western blot analyses, 150,000 GIST‑T1 cells were seeded per well in 12‑well plates (Sarstedt) 24 h before treatment. For PBN incubation, cells were exposed to 400 µL of fresh, pre‑warmed serum-free culture medium supplemented with 100 µL of siCDK4-loaded PBN formulations at the indicated concentrations. Cells treated with 100 µL of 5% glucose served as the non‑treated control condition (NT). After 1.5 h of incubation at 37 °C, 500 µL of complete culture medium supplemented with 20% FBS was added to each well to reach a final FBS concentration of 10%, without removing the PBN formulations. Cells were then incubated for an additional 24 h prior to cell lysis.

Following treatment, cells were lysed on ice using RIPA buffer composed of 50 mM Tris‑HCl (pH 8.0), 150 mM NaCl, 1% Triton X‑100, and 0.1% SDS (Sigma‑Aldrich), supplemented with protease inhibitors (SigmaFAST, Sigma‑Aldrich). Cells were incubated with 130 µL of lysis buffer for 5 min on ice, scraped, and transferred to 1.5 mL microcentrifuge tubes. Lysates were further incubated on ice for 5 min and centrifuged at 13,500 rpm for 5 min at 4 °C. The supernatants were collected, and protein concentrations were determined using the Pierce™ BCA Protein Assay (Thermo Fisher Scientific).

Equivalent amounts of protein were separated by SDS‑PAGE using 4-20% Mini‑PROTEAN TGX™ precast gels (Bio‑Rad) and transferred onto Trans‑Blot® Turbo Mini PVDF membranes (Bio‑Rad). Membranes were blocked with TBS buffer (pH 8) containing 0.2% Tween 20 (Sigma-Aldrich) and 5% bovine serum albumin (BSA, Fisher Scientifics) and incubated overnight at 4 °C with the appropriate primary antibodies diluted 1:1,000 in blocking buffer. The following rabbit monoclonal antibodies were used: anti‑CDK4 (D9G3E) and anti‑Vinculin (E1E9V), the latter serving as a loading control. After three washes in TBS containing 0.2% Tween‑20, membranes were incubated for 1 h at room temperature with HRP‑conjugated anti‑rabbit IgG secondary antibodies (1:1,000). All antibodies were obtained from Cell Signaling Technology.

Protein bands were visualized using the Pierce™ ECL Plus Western Blotting Substrate (Thermo Fisher Scientific) and imaged with a Sapphire RGB & NIR Biomolecular Imager (Azure Biosystems). Band intensities were quantified using Fiji/ImageJ software.

**Cell cytotoxicity measurement.**

Potent PBN‑induced cytotoxicity was assessed using the Cytotoxicity Detection Kit^PLUS^ (LDH; Sigma‑Aldrich), according to the manufacturer’s instructions. The assay was performed as previously described.^5^

**Confocal microscopy.**

For confocal microscopy experiments, circular glass coverslips (18 mm diameter) were placed in each well of a 12‑well culture plate (Sarstedt) before cell seeding. HeLa cells were then seeded at a density of 110,000 cells per well in complete culture medium. Twenty‑four hours after seeding, the culture medium was removed and replaced with 840 µL of DMEM supplemented with 1% P/S and without FBS.

Nanoparticle formulations containing 1 µg mCHERRY pDNA were added to the cells in a volume of 160 µL, resulting in a total volume of 1 mL per well. Cells were incubated with the PBN for 1.5 h at 37 °C. Following this incubation, FBS was added to obtain a final concentration of 10%, and the PBN were maintained in the culture medium for an additional 24 h.

After transfection, cells were fixed with 2% paraformaldehyde (PFA, CliniSciences). Nuclear staining was performed using Hoechst (1 mg/mL, Sigma-Aldrich). Finally, the cells were washed with D-PBS three times and mounted on glass slides with poly-(vinyl alcohol) Mowiol™ 4–88 glycerol Tris buffer (Biovalley). Confocal images were acquired using an inverted Zeiss LSM800 microscope with an Apo 63x/1.2 W DICIII objective. To minimize fluorophore crosstalk, sequential image acquisition was applied. Acquired images were analyzed using Fiji (ImageJ) software.

**References:**

1. Belin BJ, Lee T, Mullins RD. DNA Damage Induces Nuclear Actin Filament Assembly by Formin -2 and Spire-½ That Promotes Efficient DNA Repair. [Corrected]. *eLife* 2015;4:e07735, DOI: 10.7554/eLife.07735.

2. Konate K, Lindberg MF, Vaissiere A, Jourdan C, Aldrian G, Margeat E, Deshayes S, Boisguerin P. Optimisation of Vectorisation Property: A Comparative Study for a Secondary Amphipathic Peptide. *Int J Pharm* 2016;509(1–2):71–84, DOI: 10.1016/j.ijpharm.2016.05.030.

3. García-Valverde A, Rosell J, Sayols S, Gómez-Peregrina D, Pilco-Janeta DF, Olivares-Rivas I, de Álava E, Maurel J, Rubió-Casadevall J, Esteve A, Gut M, Valverde C, Barretina J, Carles J, Demetri GD, Fletcher JA, Arribas J, Serrano C. E3 Ubiquitin Ligase Atrogin-1 Mediates Adaptive Resistance to KIT-Targeted Inhibition in Gastrointestinal Stromal Tumor. *Oncogene* 2021;40(48):6614–6626, DOI: 10.1038/s41388-021-02049-0.

4. Taguchi T, Sonobe H, Toyonaga S, Yamasaki I, Shuin T, Takano A, Araki K, Akimaru K, Yuri K. Conventional and Molecular Cytogenetic Characterization of a New Human Cell Line, GIST-T1, Established from Gastrointestinal Stromal Tumor. *Lab Invest* 2002;82(5):663–665, DOI: 10.1038/labinvest.3780461.

5. Konate K, Dussot M, Aldrian G, Vaissière A, Viguier V, Neira IF, Couillaud F, Vivès E, Boisguerin P, Deshayes S. Peptide-Based Nanoparticles to Rapidly and Efficiently “Wrap 'n Roll” siRNA into Cells. *Bioconjug Chem* 2019;30(3):592–603, DOI: 10.1021/acs.bioconjchem.8b00776.

**Table S1: Characterization of the WRAP5:siRNA PBNs at d0 by DLS.**

| Condition | FRR | | TFR (mL/min) | Mean size (d.nm) | | PdI |
| --- | --- | --- | --- | --- | --- | --- |
| W5:siRNA (B) | 3:1 | 1.5 | | | 53.1 ± 1.7 | 0.192 ± 0.020 |
|  |  | 5 | | | 55.1 ± 2.4 | 0.215 ± 0.026 |
|  |  | 8 | | | 53.1 ± 1.4 | 0.192 ± 0.017 |
| W5:siRNA (H) | 3:1 | 1.5 | | | 47.8 ± 1.2 | 0.195 ± 0.016 |
|  |  | 5 | | | 62.3 ± 9.9 | 0.260 ± 0.076 |
|  |  | 8 | | | 57.8 ± 1.5 | 0.200 ± 0.013 |
| W5:siRNA (B) | 5:1 | 1.5 | | | 57.6 ± 2.6 | 0.198 ± 0.012 |
|  |  | 5 | | | 58.2 ± 1.0 | 0.197 ± 0.023 |
|  |  | 8 | | | 59.6 ± 3.0 | 0.191 ± 0.005 |
| W5:siRNA (H) | 5:1 | 1.5 | | | 50.6 ± 0.7 | 0.184 ± 0.007 |
|  |  | 5 | | | 66.3 ± 2.8 | 0.204 ± 0.016 |
|  |  | 8 | | | 66.6 ± 2.5 | 0.212 ± 0.016 |

*Footnotes: All WRAP5:siRNA complexes were formed at MR = 20 using a siRNA concentration of 500 nM. All formulations were performed in a filtered (0.22 µm) aqueous solution of 5% glucose (final volume of 0.5 mL). DLS measurements were performed on the same day of formulation (d0). n = 2 independent formulations (3 measures per run). PdI = Polydispersity Index. B = Baffle. H = Herringbone.*

**Table S2: Characterization of the WRAP5:pDNA PBNs at d0 by DLS.**

| Condition | FRR | TFR (mL/min) | Mean size (d.nm) | PdI |
| --- | --- | --- | --- | --- |
| W5:pDNA (B) | 3:1 | 1.5 | 50.3 ± 1.5 | 0.188 ± 0.016 |
|  |  | 5 | 49.4 ± 1.5 | 0.193 ± 0.018 |
|  |  | 8 | 48.4 ± 1.6 | 0.201 ± 0.043 |
| W5:pDNA (H) | 3:1 | 1.5 | 53.2 ± 2.4 | 0.190 ± 0.011 |
|  |  | 5 | 52.7 ± 1.0 | 0.203 ± 0.019 |
|  |  | 8 | 48.9 ± 0.8 | 0.205 ± 0.027 |
| W5:pDNA (B) | 5:1 | 1.5 | 51.4 ± 0.7 | 0.228 ± 0.050 |
|  |  | 5 | 48.8 ± 2.7 | 0.309 ± 0.087 |
|  |  | 8 | 100.5 ± 60.5 | 0.241 ± 0.052 |
| W5:pDNA (H) | 5:1 | 1.5 | 56.0 ± 2.3 | 0.184 ± 0.021 |
|  |  | 5 | 51.0 ± 5.7 | 0.216 ± 0.095 |
|  |  | 8 | 50.9 ± 1.5 | 0.233 ± 0.031 |

*Footnotes: All WRAP5:pDNA complexes were formulated at CR = 3 using a pDNA concentration of 5.2 nM. All formulations were performed in a filtered (0.22 µm) aqueous solution of 5% glucose (final volume of 0.5 mL). DLS measurements were performed on the same day of formulation (d0). n = 2 independent formulations (3 measures per run). PdI = Polydispersity Index. B = Baffle. H = Herringbone.*

**Table S3: Characterization of the WRAP5:siRNA PBNs at d7 by DLS.**

| Condition | FRR | TFR (mL/min) | Mean size (d.nm) | PdI |
| --- | --- | --- | --- | --- |
| W5:siRNA (B) | 3:1 | 1.5 | 77.6 ± 3.9 | 44.7 ± 3.8 |
|  |  | 5 | 81,9 ± 1.9 | 36.6 ± 10.5 |
|  |  | 8 | 77.9 ± 3.9 | 40.9 ± 10.4 |
| W5:siRNA (H) | 3:1 | 1.5 | 95.7 ± 7.2 | 45.1 ± 13.8 |
|  |  | 5 | 94.8 ± 12.5 | 41.9 ± 11.8 |
|  |  | 8 | 80.0 ± 1.3 | 37.3 ± 11.1 |
| W5:siRNA (B) | 5:1 | 1.5 | 77.6 ± 3.9 | 44.7 ± 3.8 |
|  |  | 5 | 81.9 ± 1.8 | 36.6 ± 10.5 |
|  |  | 8 | 77.9 ± 3.9 | 40.9 ± 10.4 |
| W5:siRNA (H) | 5:1 | 1.5 | 95.7 ± 7.2 | 45.1 ± 13.8 |
|  |  | 5 | 94.8 ± 12.5 | 41.9 ± 11.8 |
|  |  | 8 | 80.0 ± 1.3 | 37.3 ± 11.1 |

*Footnotes: All WRAP5:siRNA complexes were formed at MR = 20 using a siRNA concentration of 500 nM. All formulations were performed in a filtered (0.22 µm) aqueous solution of 5% glucose (final volume of 0.5 mL). DLS measurements were performed at 7 days post-formulation (d7). n = 2 independent formulations (3 measures per run). PdI = Polydispersity Index. B = Baffle. H = Herringbone. M = Manual.*

**Table S4: Characterization of the WRAP5:pDNA PBNs at d7 by DLS.**

| Condition | FRR | TFR (mL/min) | Mean size (d.nm) | PdI |
| --- | --- | --- | --- | --- |
| W5:pDNA (B) | 3:1 | 1.5 | 53.3 ± 1.4 | 0.147 ± 0.009 |
|  |  | 5 | 51.7 ± 1.4 | 0.146 ± 0.011 |
|  |  | 8 | 47.9 ± 0.6 | 0.145 ± 0.017 |
| W5:pDNA (H) | 3:1 | 1.5 | 53.6 ± 3.1 | 0.160 ± 0.012 |
|  |  | 5 | 50.3 ± 1.8 | 0.167 ± 0.026 |
|  |  | 8 | 48.9 ± 0.6 | 0.131 ± 0.013 |
| W5:pDNA (B) | 5:1 | 1.5 | 51.7 ± 3.6 | 0.145 ± 0.014 |
|  |  | 5 | 46.4 ± 0.9 | 0.213 ± 0.023 |
|  |  | 8 | 44.7 ± 0.7 | 0.196 ± 0.038 |
| W5:pDNA (H) | 5:1 | 1.5 | 59.8 ± 1.5 | 0.174 ± 0.032 |
|  |  | 5 | 50.9 ± 1.2 | 0.171 ± 0.021 |
|  |  | 8 | 49.3 ± 1.2 | 0.142 ± 0.012 |

*Footnotes: All WRAP5:pDNA complexes were formulated at CR = 3 using a pDNA concentration of 5.2 nM. All formulations were performed in a filtered (0.22 µm) aqueous solution of 5% glucose (final volume of 0.5 mL). DLS measurements were performed at 7 days post-formulation (d7). n = 2 independent formulations (3 measures per run). PdI = Polydispersity Index. B = Baffle. H = Herringbone.*

**Table S5: Characterization of the WRAP5:siRNA “upscale” PBNs at d55 by DLS.**

| Condition | FRR | TFR (mL/min) | Mean size (d.nm) | | PdI | |
| --- | --- | --- | --- | --- | --- | --- |
| W5:siRNA (B) | 1:1 | 5 | 149.5 ± 4.9 | 0.374 ± 0.020 | |  |
|  | 3:1 |  | 119.9 ± 4.3 | 0.319 ± 0.042 | |  |
|  | 5:1 |  | 152.4 ± 2.0 | 0.409 ± 0.018 | |  |
| W5:siRNA (H) | 1:1 | 5 | 177.6 ± 3.0 | 0.425 ± 0.012 | |  |
|  | 3:1 |  | 135.9 ± 0.9 | 0.316 ± 0.046 | |  |
|  | 5:1 |  | 191.6 ± 5.6 | 0.425 ± 0.021 | |  |
| Upscale-W5:siRNA (B) | 1:1 | 5 | 169.5 ± 3.5 | 0.361 ± 0.038 | |  |
| Upscale-W5:siRNA(H) | 1:1 | 5 | 111.5 ± 1.0 | 0.189 ± 0.004 | |  |
| Upscale-W5:siRNA (M) | 1:1 | - | 100.1 ± 2.6 | 0.245 ± 0.008 | |  |
| W5:siRNA (M) fresh | 1:1 | - | 70.9 ± 1.88 | 0.332 ± 0.024 | |  |

*Footnotes: All WRAP5:siRNA complexes were formed at MR = 20 using a siRNA concentration of 500 nM. All formulations were performed in a filtered (0;22 µm) aqueous solution of 5% glucose. DLS measurements were performed at 55 days post-formulation (d55). Additionally, a freshly manually prepared WRAP5:siRNA formulation was measured for the transfection assay shown in Figure 2B. n = 2 independent formulations (3 measures per run). PdI = Polydispersity Index. B = Baffle. H = Herringbone. M = Manual. Formulations were performed at a final volume of 0.5 mL or at 1.5 mL for the upscale conditions.*

**Table S6: Characterization of the WRAP5:pDNA PBNs at d48 and d70 by DLS.**

| Time (days) | Condition | FRR | TFR (mL/min) | Mean size (d.nm) | PdI |
| --- | --- | --- | --- | --- | --- |
| d48 | W5:pDNA (B) | 1:1 | 1.5 | 52.5 ± 0.5 | 0.156 ± 0.011 |
|  |  |  | 5 | 49.3 ± 2.5 | 0.191 ± 0.034 |
|  |  |  | 8 | 47.9 ± 1.4 | 0.184 ± 0.027 |
|  | W5:pDNA (H) | 1:1 | 1.5 | 56.6 ± 1.6 | 0.180 ± 0.011 |
|  |  |  | 5 | 50.0 ± 1.0 | 0.177 ± 0.006 |
|  |  |  | 8 | 99.2 ± 2.2 | 0.256 ± 0.071 |
|  | W5:pDNA (B) | 3:1 | 1.5 | 50.3 ± 1.5 | 0.188 ± 0.016 |
|  |  |  | 5 | 49.4 ± 1.5 | 0.193 ± 0.018 |
|  |  |  | 8 | 47.3 ± 0.3 | 0.201 ± 0.043 |
|  | W5:pDNA (H) | 3:1 | 1.5 | 53.2 ± 2.4 | 0.190 ± 0.011 |
|  |  |  | 5 | 49.3 ± 2.3 | 0.203 ± 0.019 |
|  |  |  | 8 | 48.9 ± 0.8 | 0.205 ± 0.027 |
|  | W5:pDNA (B) | 5:1 | 1.5 | 51.4 ± 0.7 | 0.228 ± 0.050 |
|  |  |  | 5 | 48.8 ± 2.7 | 0.309 ± 0.087 |
|  |  |  | 8 | 100.5 ± 60.5 | 0.241 ± 0.052 |
|  | W5:pDNA (H) | 5:1 | 1.5 | 56.0 ± 2.3 | 0.228 ± 0.050 |
|  |  |  | 5 | 51.0 ± 5.7 | 0.216 ± 0.095 |
|  |  |  | 8 | 50.9 ± 1.5 | 0.233 ± 0.031 |
|  | Upscale-W5:pDNA (B) | 1:1 | 1.5 | 50.2 ± 0.2 | 0.119 ± 0.006 |
|  | Upscale-W5:pDNA(H) | 1:1 | 1.5 | 46.6 ± 0.4 | 0.120 ± 0.003 |
|  | Upscale-W5:pDNA (M) | 1:1 | - | 55.0 ± 0.3 | 0.175 ± 0.013 |
| d70 | W5:pDNA (B) | 1:1 | 1.5 | 60.0 ± 5.8 | 0.158 ± 0.014 |
|  |  |  | 5 | 56.0 ± 0.8 | 0.143 ± 0.010 |
|  |  |  | 8 | 55.3 ± 0.8 | 0.162 ± 0.026 |
|  | W5:pDNA (H) | 1:1 | 1.5 | 67.2 ± 3.7 | 0.144 ± 0.014 |
|  |  |  | 5 | 76.9 ± 23.2 | 0.150 ± 0.017 |
|  |  |  | 8 | 76.7 ± 6.9 | 0.190 ± 0.053 |
|  | W5:pDNA (B) | 3:1 | 1.5 | 49.6 ± 1.9 | 0.159 ± 0.027 |
|  |  |  | 5 | 50.8 ± 1.6 | 0.195 ± 0.021 |
|  |  |  | 8 | 47.7 ± 1.4 | 0.187 ± 0.017 |
|  | W5:pDNA (H) | 3:1 | 1.5 | 59.8 ± 6.6 | 0.144 ± 0.011 |
|  |  |  | 5 | 55.1 ± 2.1 | 0.184 ± 0.031 |
|  |  |  | 8 | 54.2 ± 2.8 | 0.149 ± 0.027 |
|  | W5:pDNA (B) | 5:1 | 1.5 | 53.4 ± 2.6 | 0.156 ± 0.015 |
|  |  |  | 5 | 86.9 ± 40.4 | 0.231 ± 0.061 |
|  |  |  | 8 | 67.7 ± 23.6 | 0.291 ± 0.049 |
|  | W5:pDNA (H) | 5:1 | 1.5 | 60.8 ± 2.4 | 0.147 ± 0.009 |
|  |  |  | 5 | 55.2 ± 3.2 | 0.163 ± 0.004 |
|  |  |  | 8 | 53.5 ± 0.9 | 0.149 ± 0.006 |
|  | Upscale-W5:pDNA (B) | 1:1 | 1.5 | 54.5 ± 0.6 | 0.141 ± 0.015 |
|  | Upscale-W5:pDNA(H) | 1:1 | 1.5 | 52.9 ± 1.0 | 0.131 ± 0.004 |
|  | Upscale-W5:pDNA (M) | 1:1 | - | 62.1 ± 0.8 | 0.156 ± 0.007 |

*Footnotes: All WRAP5:pDNA complexes were formulated at CR = 3 using a pDNA concentration of 5.2 nM. All formulations were performed in a filtered (0.22 µm) aqueous solution of 5% glucose. DLS measurements were performed at 48 days (d48) and 70 days post-formulation (d70). n = 2 independent formulations (3 measures per run). PdI = Polydispersity Index. B = Baffle. H = Herringbone. M = Manual. Formulations were performed at a final volume of 0.5 mL or at 1.5 mL for the upscale conditions.*
